# Higher levels of circulating desphospho-uncarboxylated matrix Gla protein over time are associated with worse survival: the prospective Maastricht Intensive Care COVID cohort

**DOI:** 10.1186/s40560-023-00712-0

**Published:** 2023-12-18

**Authors:** Mark M. G. Mulder, Joep Schellens, Jan-Willem E. M. Sels, Frank van Rosmalen, Anne-Marije Hulshof, Femke de Vries, Ruud Segers, Casper Mihl, Walther N. K. A. van Mook, Aalt Bast, Henri M. H. Spronk, Yvonne M. C. Henskens, Iwan C. C. van der Horst, Hugo ten Cate, Leon J. Schurgers, Marjolein Drent, Bas C. T. van Bussel

**Affiliations:** 1 Department of Intensive Care Medicine, Maastricht University Medical Centre+, Maastricht, The Netherlands; 2 Department of Anaesthesiology, Maastricht University Medical Centre+, Maastricht, The Netherlands; 3 Department of Cardiology, Maastricht University Medical Centre+, Maastricht, The Netherlands; 4 Central Diagnostic Laboratory, Maastricht University Medical Centre+, Maastricht, The Netherlands; 5 Department of Biochemistry, Cardiovascular Research Institute Maastricht, Maastricht University, Maastricht, The Netherlands; 6 Department of Radiology and Nuclear Medicine, Maastricht University Medical Centre+, Maastricht, The Netherlands; 7 Academy for Postgraduate Medical Training, Maastricht University Medical Centre+, Maastricht, The Netherlands; 8 School of Health Professions Education, Maastricht University, Maastricht, The Netherlands; 9 Department of Pharmacology and Toxicology, Maastricht University, Maastricht, The Netherlands; 10 Thrombosis Expert Centre Maastricht and Department of Internal Medicine, Maastricht University Medical Centre+, Maastricht, The Netherlands; 11 Cardiovascular Research Institute Maastricht (CARIM), Maastricht University, Maastricht, The Netherlands; 12 ILD Centre of Excellence, Department of Respiratory Medicine, St. Antonius Hospital, Nieuwegein, The Netherlands; 13 ILD Care Foundation Research Team, Ede, The Netherlands; 14 Care and Public Health Research Institute (CAPHRI), Maastricht University, Maastricht, The Netherlands

**Keywords:** Vitamin K deficiency, Matrix Gla protein, COVID-19, Intensive care, Pulmonary embolism

## Abstract

**Background:**

Extra-hepatic vitamin K-status, measured by dephosphorylated uncarboxylated matrix Gla protein (dp-ucMGP), maintains vascular health, with high levels reflecting poor vitamin K status. The occurrence of extra-hepatic vitamin K deficiency throughout the disease of COVID-19 and possible associations with pulmonary embolism (PE), and mortality in intensive care unit (ICU) patients has not been studied. The aim of this study was to investigated the association between dp-ucMGP, at endotracheal intubation (ETI) and both ICU and six months mortality. Furthermore, we studied the associations between serially measured dp-ucMGP and both PE and mortality.

**Methods:**

We included 112 ICU patients with confirmed COVID-19. Over the course of 4 weeks after ETI, dp-ucMGP was measured serially. All patients underwent computed tomography pulmonary angiography (CTPA) to rule out PE. Results were adjusted for patient characteristics, disease severity scores, inflammation, renal function, history of coumarin use, and coronary artery calcification (CAC) scores.

**Results:**

Per 100 pmol/L dp-ucMGP, at ETI, the odds ratio (OR) was 1.056 (95% CI: 0.977 to 1.141, *p* = 0.172) for ICU mortality and 1.059 (95% CI: 0.976 to 1.059, *p* = 0.170) for six months mortality. After adjustments for age, gender, and APACHE II score, the mean difference in plasma dp-ucMGP over time of ICU admission was 167 pmol/L (95% CI: 4 to 332, *p* = 0.047). After additional adjustments for c-reactive protein, creatinine, and history of coumarin use, the difference was 199 pmol/L (95% CI: 50 to 346, *p* = 0.010). After additional adjustment for CAC score the difference was 213 pmol/L (95% CI: 3 to 422, *p* = 0.051) higher in ICU non-survivors compared to the ICU survivors. The regression slope, indicating changes over time, did not differ. Moreover, dp-ucMGP was not associated with PE.

**Conclusion:**

ICU mortality in COVID-19 patients was associated with higher dp-ucMGP levels over 4 weeks, independent of age, gender, and APACHE II score, and not explained by inflammation, renal function, history of coumarin use, and CAC score. No association with PE was observed. At ETI, higher levels of dp-ucMGP were associated with higher OR for both ICU and six month mortality in crude and adjusted modes, although not statistically significantly.

## Introduction

Critical care settings are characterized by an increased risk of malnutrition and micronutrient deficiencies, including vitamin K deficiency [1]. This nutrient is essential for proper coagulation and cardiovascular health [2–4]. Emerging evidence suggests that vitamin K deficiency may be particularly relevant in patients with COVID-19 owing to its potential role in immune function and coagulopathy [5–10]. In severe COVID-19 cases, immune dysregulation and coagulopathy can lead to microvascular occlusion [11–13], multi-organ failure [14], and in turn, increased mortality [15].

Vitamin K is a fat-soluble vitamin that is necessary for the posttranslational gamma-glutamylcarboxylation of certain proteins, including several coagulation factors and matrix Gla protein (MGP) [16]. MGP is a vitamin K-dependent protein that plays a critical role in vascular health. Dephosphorylated uncarboxylated matrix Gla protein (dp-ucMGP) is a circulating biomarker that reflects the levels of inactive MGP and can be used as a marker of extra-hepatic vitamin K status. Higher levels of dp-ucMGP suggest impaired carboxylation of vitamin K-dependent proteins in the vasculature and extra-hepatic vitamin K deficiency [17].

Emerging evidence suggests that extra-hepatic vitamin K deficiency may be associated with worse outcome in COVID-19 [10, 18]. Other studies found that extra-hepatic vitamin K deficiency is associated with more severe lung injury and is potentially linked to thrombotic complications in COVID-19 [6, 9]. Furthermore, it has been associated with more inflammation in these patients [5, 8].

Extra-hepatic vitamin K is essential for proper coagulation and cardiovascular health, and extra-hepatic vitamin K deficiency has been associated with cardiovascular morbidity and development of severe lung injury in COVID-19 patients [6, 9, 10, 18]. Therefore, measuring dp-ucMGP levels over the trajectory of ICU admission in COVID-19 patients may provide insight into the potential association between extra-hepatic vitamin K deficiency and clinical outcomes.

The aim of this study was to investigate whether dp-ucMGP levels during ICU admission are associated with worse clinical outcomes, including thrombotic events and mortality, in critically ill COVID-19 patients. In addition, we investigated whether dp-ucMGP levels reflect cardiovascular morbidity and are associated with the development of severe lung injury.

## Methods

The manuscript was written following the STrengthening the Reporting of Observational studies in Epidemiology (STROBE) guidelines [19].

### Study population

The Maastricht Intensive Care COVID (MaastrICCht) cohort is a prospective cohort of patients with confirmed COVID-19 admitted to the ICU of the Maastricht University Medical Centre (MUMC +). The design has been described extensively elsewhere [20] and includes comprehensive serial hemostasis and coagulation phenotyping [21, 22]. The local institutional review board (Medisch Ethische Toetsingscomissie (METC) 2020-1565/300523) of the MUMC + approved the study, which was performed based on the regulations of Helsinki. The study is registered in International Clinical Trials Registry Platform (NL8613). This study included all participants with respiratory insufficiency requiring mechanical ventilation and at least one real-time polymerase chain reaction (RT-PCR) positive for SARS-CoV-2 RNA and a chest CT scan strongly suggestive of SARS-CoV-2 infection, based on a CORADS-score of 4–5 scored by a radiologist [23, 24]. Participants were followed until they either died in the ICU or were discharged from ICU. A comprehensive and uniform set of clinical, physiological, and laboratory variables was collected daily, reducing the chance of missing data. In addition, when patients were not available for blood sampling or laboratory testing failed, the measurement would be rescheduled for the next blood withdrawal.

### Clinical, physiological variables

Variable collection on the ICU for COVID-19 was standardized as described extensively elsewhere [20]. Medical history of cardiovascular disease (defined as congestive heart failure, myocardial infarction, or peripheral vascular disease) was scored on ICU admission. APACHE-II score on ETI and SOFA score during ICU stay were calculated [14]. Coronary artery calcium (CAC) scores were measured within the MaastrICCht cohort, which was described in more detail elsewhere [25]. Patients were classified with or without a clinical PE as follows; in patients with a clinical suspected PE, computed tomography pulmonary angiography (CTPA) was used diagnostically. CTPA was performed in a supine position after intravenous injection of individually adapted contrast media volume (iopromide 300 mg iodine; Ultravist, Bayer Healthcare, Berlin, Germany) based on body weight and kVp settings on a second or third-generation dual source CT scanner (Somatom Definition Flash, Force; Siemens Healthineers, Forchheim Germany). The image quality of all CT scans was judged sufficient to evaluate the presence of PE or thrombosis (central, lobular, segmental, or sub-segmental) [26]. Patients in whom CTPA excluded PE were classified as not having clinical PE. The occurrence of deep venous thrombosis (DVT) diagnosed by ultrasound was recorded within the cohort, but was not considered as the majority of the patients underwent CTPA at ICU admission.

### Six months follow-up

Information regarding the six months mortality after endotracheal intubation (ETI) was collected. This was done by identification of the last medical contact consisting of: a consultation in our hospital or hospitalisation, a visit to the emergency room, imaging diagnostics, surgery or the laboratory measurement of a blood sample drawn. When patients had died during the six month follow-up period, this information was collected. Patients who had been transferred to other hospitals, were followed-up by contacting the patients themselves or their general practitioners.

### Enteral nutrition

Enteral nutrition in all ICU admitted patients, who were suspected to be unable to ingest oral food within the first 48 h of ICU admission, has been started via naso-gastric tube. Fresubin 1200 (including 10 μg vitamin K /100ml) was the standard nutrition and was prescribed in a weight adjusted dose [27]. The adjusted feeding dose was calculated as follows: day 1; 5ml/kg/day, day 2; 10 ml/kg/day, day 3; 15ml/kg/day, and maximal dosage on day 4; 20ml/kg/day [28].

### Anticoagulation

All ICU admitted patients received intermediate doses of thromboprophylaxis: Nadroparin 5700, 7600, and 11,400 IU for respectively < 70, 70–90, and > 90 kg [29]. Patients who required therapeutic anticoagulants before hospital admission were started on therapeutic low molecular weight heparin (LMWH) upon ICU admission. In addition, vitamin K antagonists and direct oral anticoagulants (DOACs) were switched to therapeutic LMWH. Patients on extracorporeal membrane oxygenation (ECMO) or continuous renal replacement therapy (CRRT) received unfractionated heparin (UFH), guided by guidelines based on aPTT (heparin therapeutic range (HTR) 50-80s) and anti-Xa (HTR 0.3 – 0.7 IU/mL) measurements [30].

### dp-ucMGP sub-cohort

**Fig. 1:**
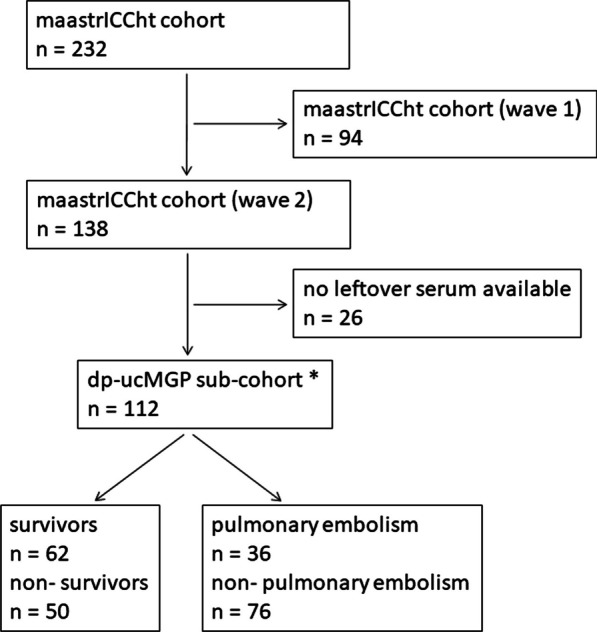
Flowchart patient population. Wave 2 patients had standard CTPA on ICU admission. *MaastrICCht* Maastricht Intensive Care COVID, *dp-ucMGP* desphospho-uncarboxylated matrix Gla protein, *dp-ucMGP was measured to determine extra hepatic vitamin K status in a sub-cohort of the Maastricht Intensive Care COVID cohort

Two-hundred and thirty-two patients were enrolled in the MaastrICCht cohort from March 25th 2020, until April 13th, 2021. We included patients in the present investigation who had a chest CT scan as part of standard care. The chest CT scan was introduced as standard of care in our hospital during the pandemic to rule out pulmonary embolism, at ICU admission, and was done in each patient. To rule out any selective information bias on coronary calcium, which is important within the pathophysiological framework under investigation, ninety-four patients enrolled early during the COVID-19 pandemic were excluded as they had no standard chest CT scan [31]. Of the total of 232 cohort patients, dp-ucMGP as therefore not measured in the initial 94 patients. Hundred and thirty-eight patients were enrolled in the MaastrICCht cohort, during the second COVID-19 wave, from September 26th, 2020, until April 13th, 2021. Of those hundred thirty eight, a hundred and twelve patients had serial citrate plasma stored to measure dp-ucMGP. No leftover citrate plasma was available in the remaining twenty-six patients, which were excluded (Fig. 1). Timing from ETI allows for a fairer comparison between vitamin K status and the disease course severity, where disease severity is defined as the need for mechanical ventilation in the ICU due to COVID-19. From September 29th onwards, additional dp-ucMGP assays were performed at Monday and Thursday in the morning, in leftover citrate plasma, for all included MaastrICCht cohort patients. Patients who were in the ICU before September 29th or were transported from another hospital after ETI were also included, starting dp-ucMGP measurements from admission from September 29th onwards. This means that the inclusion of patients could vary between the first till the fourth week after ETI. This design has been applied and described more extensively elsewhere [20].

### Blood withdrawal and preparation and laboratory analysis

Daily arterial blood samples from all patients were collected from an arterial line in 7.2 mg K2 EDTA (4.0 mL), serum, or 3.2%(w/v) sodium citrate Vacutainer blood collection tubes (Becton Dickinson, Plymouth, UK). Platelet-poor plasma (PPP) was obtained using two subsequent centrifugation steps: initial centrifugation of 2490*g* for 5 min, followed by 10,000*g* for 10 min. Circulating dp-ucMGP levels were determined in citrate plasma using the commercially available IVD chemiluminescent InaKif MGP assay on the IDS-iSYS system (IDS, Boldon, United Kingdom) as previously described [32]. The within-run and total precision of this assay were 0.8–6.2% and 3.0–8.2%, respectively. The assay measuring range is between 200 and 12,000 pmol/L and was found to be linear up to 11,651 pmol/L. dp-ucMGP values < 400 pmol/L are in the normal healthy range and values > 400 pmol/L reflect vitamin K deficiency [17].

### Statistical analyses

The data were analyzed with R version 3.6.1. As appropriate, the sample characteristics were described using median and interquartile range (IQR) or percentage. In addition, Pearson’s chi-square test, Mann Witney u test, or Kruskal–Wallis test were performed to compare characteristics. We described the association between serial dp-ucMGP measurements during ICU admission and two outcome variables. First, the cohort participants were categorized based on ICU survivors and ICU non-survivors. Then, we used linear mixed-effects regression with a random intercept and random slope for time to compute average differences in dp-ucMGP over time and differences in the slope over time between both groups. If the slope did not differ, average differences over time are reported only. We computed unadjusted group differences for dp-ucMGP (Model 1). In model 2, we adjusted model 1 for age, gender, and APACHE-II score. In model 3, we additionally adjusted model 2 for C-reactive protein (at ETI), creatinine (at ETI) and history of coumarin use. In addition, to investigate whether differences in dp-ucMGP between ICU survivors and ICU non-survivors were independent of pre-existing cardiovascular disease, we additionally adjusted model 2 for CAC-score (model 4) as more CAC reflects worse cardiovascular disease [31, 32]. Finally, we categorized patients based on the occurrence of PE (CTPA positive vs. CTPA negative) and repeated models 1–3 above. We report regression coefficients β with 95% confidence intervals (95% CI) and considered a *p*-value < 0.05 statistically significant. In addition, we analysed the associations between dp-ucMGP, per 100 pmol/L, and ICU mortality and six-months mortality, in crude (model 1) and adjusted models (adjusting for age, gender, APACHE II score and c-reactive protein, creatinine, and history of coumarin use (model 2)).

## Results

### Patient characteristics

**Fig. 2:**
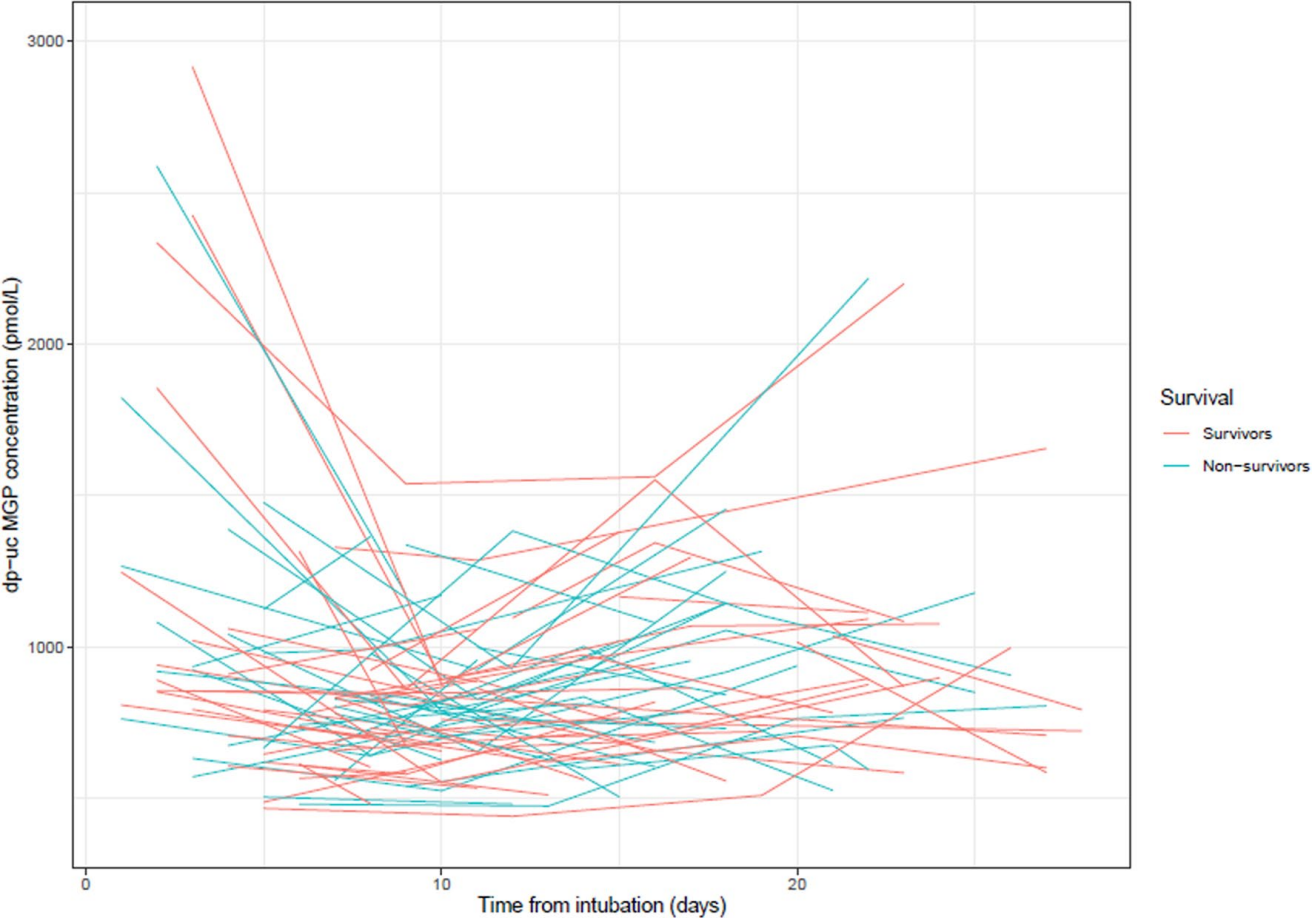
Evolution of desphospho-uncarboxylated matrix Gla protein (dp-ucMGP) levels (pmol/L) for individual patients, during intensive care unit (ICU) stay, for ICU survivors and ICU non-survivors suffering from COVID-19

Of 112 mechanically ventilated patients, the median [IQR] age was 65 [60–71] years, and 78% were men. Body Mass Index (BMI) was 27.7 [25.4–31.1] (Kg/m^2^). Before ETI, 27 (24%) participants had cardiovascular disease, 26 (23%) had chronic pulmonary disease, and 5 patients (5%) had a history of coumarin use. CAC scores were measured in 79 (69%) patients. On the moment of ETI, median [IQR] C-reactive protein was 94 [45–154] (mg/L), leukocytes 10.4 [7.2–12.7] (x10E9/L), creatinine 74 [59–97] (µmol/l), alkaline phosphatase 76 [58–108] (IU/L), lactate dehydrogenase was 469 [361–650] (U/L), pt 11.5 [10.9–12.2] (sec), aPTT 30 [27–35] (sec), d-dimer 2103 (ng/mL) [1096–7309], thrombocytes 257 (×10E9/L) [177–334] and APACHE II score 15 [13–18]. During the ICU stay, 8 patients (7%) underwent renal replacement therapy, and 6 patients (5%) underwent extracorporeal membrane oxygenation (ECMO). The number of ICU survivors was 63 (56%). Age (68 vs. 63 years) and APACHE II score (16 vs. 14) were significantly higher in ICU non-survivors than in ICU survivors, respectively (*p* < 0.05). BMI (27 vs. 28.7 kg/m^2^), as well as the leukocytes (9.1 vs. 11.5 × 10E9/L) and thrombocytes (220 vs. 288 × 10E9/L), which were significantly lower in ICU non-survivors (*p* < 0.05). The number of patients with chronic pulmonary disease (7 vs. 19) was significantly lower in ICU non-survivors as well (*p* < 0.05). 36 patients (32%) were diagnosed with PE (Table 1). Follow-up was 4 weeks, and the number of analyzed samples per week varied between 25 and 89. The level of dp-ucMGP and SOFA score did not significantly differ between the various time points during follow-up for ICU survivors and ICU non-survivors (Table 2). Figure 2 shows the individual trajectories of measured dp-ucMGP levels (pmol/L) for ICU survivors and ICU non-survivors.

**Table 1:** Summary of the patient characteristics

General	Total	Survivors	Non-survivors	*p*-value	PE-	PE +	*p*-value
Number	112	62	50	NA	76	36	NA
Male, n (%)	87 [77.6]	48 [77]	39 [78]	0.942	55 [72]	32 [88]	0.050
Age (year), median [IQR]	65 [60–71]	63 [56–69]	68 [63–73]	0.001	65 [59–72]	66 [60–73]	0.572
Body mass index (Kg/m.^2^), median [IQR]	27.7 [25.4–31.1]	28.7 [26.2–32.4]	27 [24.7–29.4]	0.016	27.8 [25.2–31.4]	26.9 [25.4–29.7]	0.334
Medical History							
Cardiovascular disease, yes, *n* [%]	27 [24.1]	13 [21]	14 [28]	0.387	19 [25]	8 [22]	0.748
Chronic pulmonary disease, *n* [%]	26 [23.2]	19 [30]	7 [14]	0.038	18 [23.6]	8 [22]	0.864
History of coumarin use, *n* [%]	5 [4.5]	3 [4.8]	2 [4]	0.831	5 [6.5]	0 [0]	0.115
Laboratory variables							
C-reactive protein (mg/L), median [IQR]	94 [45–154]	76 [45–151]	100 [44–168]	0.484	83 [47–146]	118 [36–188]	0.448
Leucocytes (x10E9/L), median [IQR]	10.4 [7.2–12.7]	11.5 [8.3–14.5]	9.1 [6.5–11.5]	0.004	9.8 [6.9–12.5]	11 [8.5–13]	0.101
Creatinine (µmol/l), median [IQR]	74 [59–97]	71 [59–97]	76 [60–98]	0.824	74 [60–101]	75 [58–92]	0.906
Alkaline phosphatase (IU/L), median [IQR]	76 [58–108]	72 [58–107]	83 [58.5–113]	0.355	73 [59–103]	81 [55–137]	0.168
Lactate dehydrogenase (U/L), median [IQR]	469 [361–650]	465 [353–671]	498 [365–597]	0.792	468 [356–644]	492 [361–653]	0.965
Prothrombin time (sec), median [IQR]	11.5 [10.9–12.2]	11.5 [10.9–12.4]	11.5 [10.8–12]	0.690	11.5 [10.9–12.5]	11.7 [11.0–12.0]	0.748
Activated partial thromboplastin time (sec), median [IQR]	30 [27–35]	29 [26–33]	30 [28–39]	0.089	29 [27–35]	31 [27–37]	0.363
D–dimer (ng/mL), median [IQR]	2103 [1096–7309]	2129 [1056–7752]	2075 [1167–7591]	0.841	2089 [1050–6965]	3028 [1271–9101]	0.507
Thrombocytes (x10E9/L), median [IQR]	257 [177–334]	280 [227–342]	220 [146–316]	0.006	244 [175–331]	294 [177–347]	0.132
ICU events							
APACHE II score at ETI, median [IQR]	15 [13–18]	14 [11–17]	16 [14–19]	0.035	14.5 [12–18]	15 [13–18]	0.630
Length of ICU stay (days), median [IQR]	14 [10–24]	14 [9–26]	18 [11–24]	0.654	14 [9–21]	18 [13–31]	0.010
Duration of ventilation (days), median [IQR]	11 [6–20]	10 [7–23]	13 [5–16]	0.682	9 [6–16]	16 [8–36]	0.060
RRT during ICU stay, *n* [%]	8 [7.1]	3 [4.8]	5 [10]	0.292	6 [7.9]	2 [5.5]	0.653
ECMO during ICU stay, *n* [%]	6 [5.3]	2 [3.2]	4 [8]	0.265	3 [4]	3 [8.3]	0.336
PE, *n* [%]	36 [32]	17 [9]	19 [38]	0.233	NA	NA	NA
ICU mortality, *n* [%]	50 [44]	NA	NA	NA	31 [41]	19 [52]	0.233
6-months mortality, *n* [%]	53 [47]	3 [4.8]	50 [100]	0.001	34 [45]	19 [52]	0.431

*IQR* interquartile range, *NA* not applicable, *ICU* intensive care unit, *PE +*  pulmonary embolism approved, *PE−* pulmonary embolism excluded, *APACHE II score* Acute Physiology and Chronic Health Evaluation II score, *RRT* renal replacement therapy, *ECMO* extracorporeal membrane oxygenation, *ETI* endotracheal intubation, Pearson’s chi-square test was used for nominal data, Mann witney u test was used for non-parametric data, *p*-value < 0.05

**Table 2:** Number of COVID-19 patients, citrate sampling sample dates, desphospho-uncarboxylated matrix Gla protein (dp-ucMGP) levels (pmol/L), routine laboratory measurements, and Sequential Organ Failure Assessment (SOFA) scores per week of intensive care unit (ICU) admission starting from endotracheal intubation for ICU survivors and ICU non-survivors

Variables	0		1		2		3		*p*-value	
	Survivor	Non-survivor	Survivor	Non-survivor	Survivor	Non-survivor	Survivor	Non-survivor	Survivor	Non-survivor
Serum Samples, n	48	41	40	36	25	24	18	7	NA	NA
Number of days (days), median [IQR]	4 [2–6]	5 [3–6]	10 [9–11]	10 [9–12]	16 [15–19]	18 [16–19]	24 [23–27]	24 [22–26]	NA	NA
dp-ucMGP (pmol/L), median [IQR]	821 [649-1022]	920 [714-1227]	724 [609-874]	835 [642-998]	865 [697-1118]	928 [679-1134]	887 [686-1087]	850 [767-1180]	0.070	0.472
SOFA score, median [IQR]	10 [7–11]	11 [10–12]	8 [7–11]	12 [10–12]	7 [7–10]	12 [11, 12]	8 [7–12]	12 [10–12]	0.472	0.805

*IQR* interquartile range, *NA* not applicable, Kruskal–Wallis test was used for non-parametric data, *p*-value < 0.05

### Associations between dp-ucMGP at ETI and both ICU and six months mortality

Per 100 pmol/L dp-ucMGP at ETI the odds ratio for ICU mortality was 1.056 (95% CI: 0.977–1.141) (crude). After adjustment for age, gender, APACHE II score, c-reactive protein (at ETI), creatinine (at ETI), and history of coumarin use, the odds ratio for ICU mortality was 1.072 (95% CI: 0.985–1.166). Per 100 pmol/L dp-ucMGP at ETI the odds ratio for six months mortality was 1.059 (95% CI: 0.976–1.059) (crude). After adjustment for age, gender, APACHE II score, c-reactive protein (at ETI), creatinine (at ETI), and history of coumarin use, the odds ratio for six months mortality was 1.069 (95% CI: 0.984–1.163) (Table 3).

**Table 3:** Association between desphospho-uncarboxylated matrix Gla protein (dp-ucMGP) levels at the moment of ETI and ICU mortality and six months mortality

	ICU mortality			Six months mortality		
	Odds ratio	95% confidence interval	*p*-value	Odds Ratio	95% confidence interval	*p*-value
dp-ucMGP (model 1)	1.056	0.977–1.141	0.172	1.059	0.976–1.059	0.170
dp-ucMGP (model 2)	1.072	0.985–1.166	0.109	1.069	0.984–1.163	0.115

Data are OR with their 95% CI based on logistic regression analyses and show the association between dephosphorylated uncarboxylated matrix Gla protein per 100pmol/L at ETI and mortality, for ICU mortality and six months mortality respectively. Model 1 = crude, Model 2 = adjusted for age + gender + APACHE II score + c-reactive protein (at ETI) + creatinine (at ETI) + history of coumarin use. *dp-ucMGP* dephosphorylated uncarboxylated matrix Gla protein, *ICU* intensive care unit, ETI = endotracheal intubation

### dp-ucMGP and ICU mortality

The average dp-ucMGP level for ICU survivors was 866 pmol/L (95% CI: 758 to 974) and 1025 pmol/L (95% CI: 906 to 1145) for ICU non-survivors. For ICU non-survivors, the average (95% CI) dp-ucMGP level over time was 159 pmol/L (95% CI: − 2 to 321, *p* = 0.054) higher compared to the ICU survivors (Table 4, model 1). After adjustment for age, gender, and APACHE II score, the average level of (95% CI) dp-ucMGP over time was 167 pmol/L (95% CI: 4 to 332 *p* = 0.047) higher for ICU non-survivors compared to the ICU survivors (Table 4, model 2).

**Table 4:** Linear mixed-effects regression for desphospho-uncarboxylated matrix Gla protein (dp-ucMGP) levels (pmol/L) and intensive care unit (ICU) mortality

Model	dp-ucMGP (pmol/L)	CI (95%) (pmol/L)	*p*-value
Model 1: crude			
ICU survivors (reference)	Ref.	Ref.	Ref.
ICU non-survivors	159	− 2–321	0.054
Model 2: model 1 + age + gender + APACHE II score			
ICU survivors (reference)	Ref.	Ref.	Ref.
ICU non-survivors	167	4–332	0.047
Model 3: model 2 + c-reactive protein (at ETI) + creatinine (at ETI) + history of coumarin use			
ICU survivors (reference)	Ref.	Ref.	Ref.
ICU non-survivors	199	50–346	0.010
Model 4: model 2 + CAC score			
ICU survivors (reference)	Ref.	Ref.	Ref.
ICU non-survivors	213	3–422	0.051

*CI* confidence interval, *APACHE II score* Acute Physiology, and Chronic Health Evaluation II score, *CAC score* Coronary artery calcifications scores, *ETI* endotracheal intubation, Group differences were calculated compared to the reference, *p*-value < 0.05

### The role of inflammation, creatinine, history of coumarin use and CAC

**Fig. 3:**
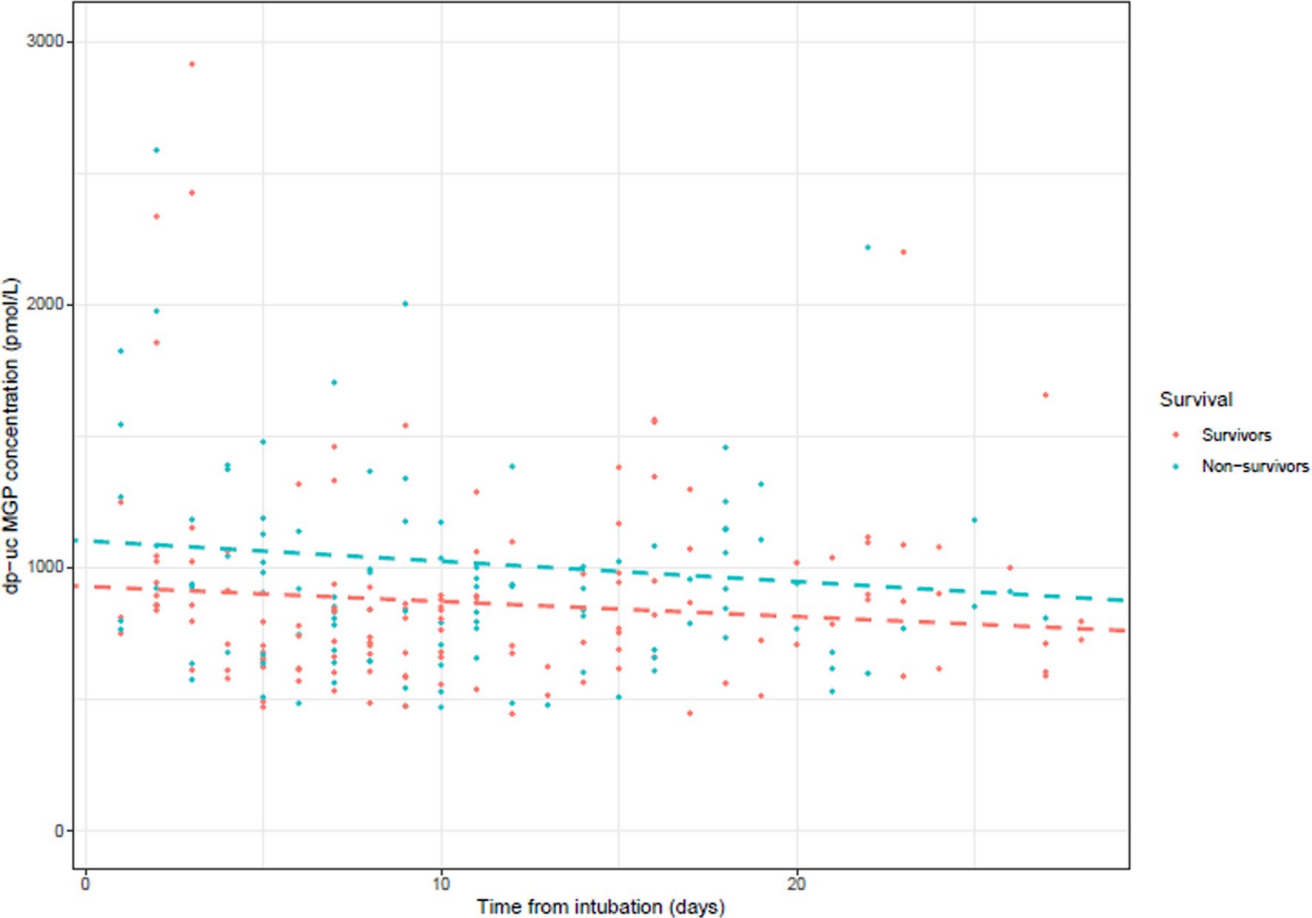
Evolution of desphospho-uncarboxylated matrix Gla protein (dp-ucMGP) levels (pmol/L) during intensive care unit (ICU) stay, for ICU survivors and ICU non-survivors suffering from COVID-19

Additional adjustments for C-reactive protein, creatinine, and history of coumarin use did not change the difference (Table 4, model 3), which became somewhat greater, (i.e., 213 95% CI (-3 to 422)) after adjustment for CAC scores, between ICU survivors and ICU non-survivors (Table 4, model 4). Differences in average level of dp-ucMGP levels between ICU survivors and ICU non-survivors remain stable over time (*p* = 0.138) (Fig. 3).

### dp-ucMGP and pulmonary embolism in the ICU

**Fig. 4:**
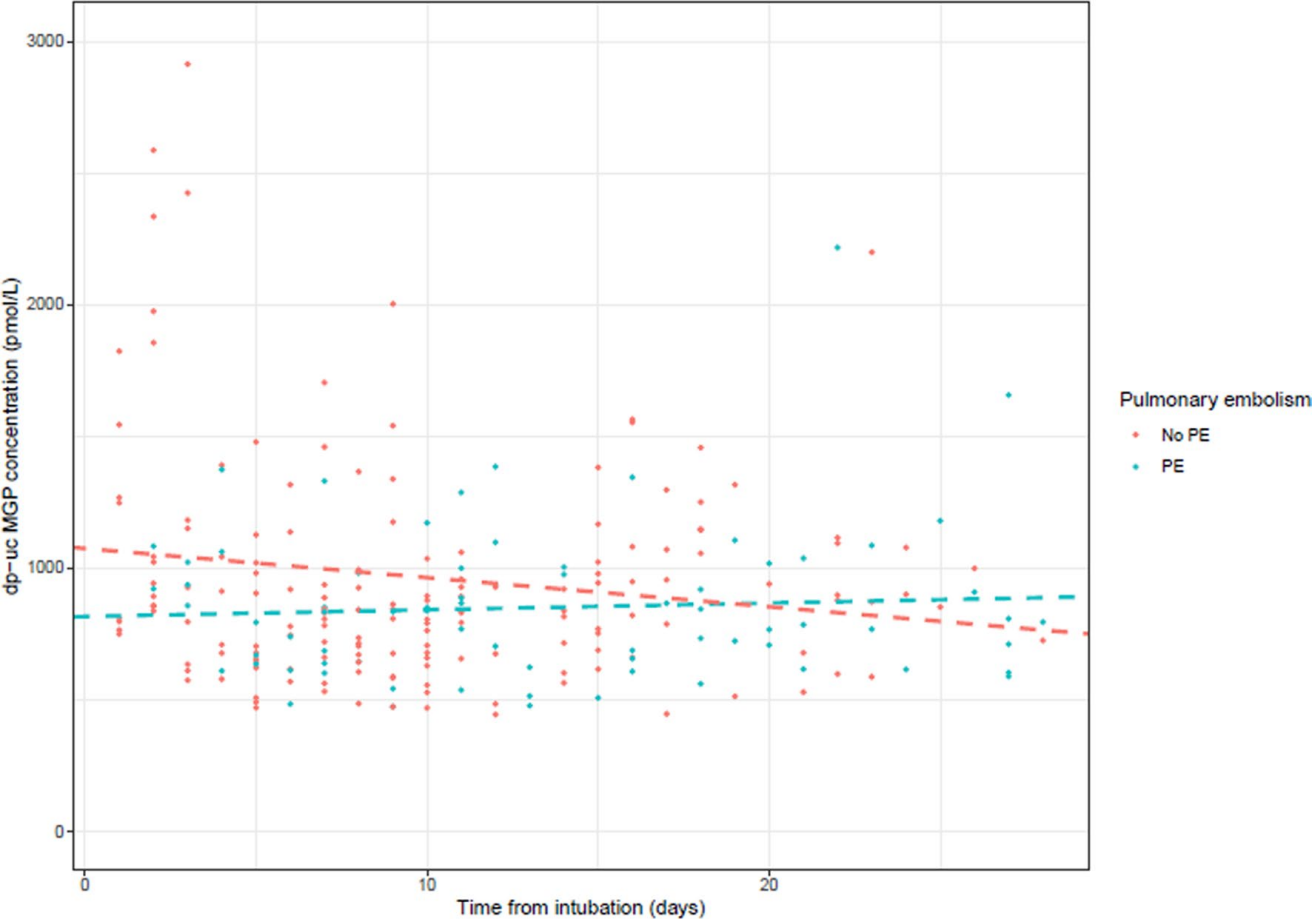
Evolution of desphospho-uncarboxylated matrix Gla protein (dp-ucMGP) levels (pmol/L), during intensive care (ICU) stay, for patients suffering from COVID-19 with approved pulmonary embolism (PE) and patients in which PE was excluded

The average dp-ucMGP level for patients without PE was 973 pmol/L (95% CI: 875 to 1072), and for patients with PE was 849 pmol/L (95% CI: 700 to 998). For patients with PE, the average (95% CI) dp-ucMGP level over time was -124 pmol/L (95% CI: -301 to 54, *p* = 0.219) lower compared to patients without PE (Table 5, model 1). Additional adjustments for age, gender, APACHE II score, C-reactive protein at ETI, creatinine at ETI, and history of coumarin use (Table 5, model 3) did not change this result. Differences in average dp-ucMGP did not change over time between patients with or without PE (*p* = 0.054), (Fig. 4).

**Table 5:** Linear mixed-effects regression for desphospho-uncarboxylated matrix Gla protein (dp-ucMGP) levels (pmol/L) and pulmonary embolism (PE) in the intensive care unit (ICU)

Model	dp-ucMGP (pmol/L)	CI (95%) (pmol/L)	*p*-value
Model 1: crude			
No pulmonary embolism (reference)	Ref.	Ref.	Ref.
Presence of pulmonary embolism	− 124	− 301–54	0.219
Model 2: model 1 + age + gender + APACHE II score			
No pulmonary embolism (reference)	Ref.	Ref.	Ref.
Presence of pulmonary embolism	− 98	− 274–77	0.274
Model 3: model 2 + c-reactive protein (at ETI) + creatinine (at ETI) + history of coumarin use			
No pulmonary embolism (reference)	Ref.	Ref.	Ref.
Presence of pulmonary embolism	− 31	− 195–133	0.712

*CI* confidence interval, *APACHE II score* Acute Physiology and Chronic Health Evaluation II score, *ETI* endotracheal intubation, Group differences were calculated compared to the reference, *p*-value < 0.05

## Discussion

This study evaluated the extra-hepatic vitamin K status throughout the disease course of COVID-19 and possible associations with PE, and mortality in ICU admitted patients.

At the moment of ETI, patients with severe COVID-19 had dp-ucMGP levels far above the reference range [17]. This study investigated the association between dp-ucMGP levels and PE in ICU admitted patients. The results showed that dp-ucMGP levels did not differ between patients with PE as compared to those without PE. Therewith, we could not confirm the suggestion that extra-hepatic vitamin K deficiency may play a role in the development of PE in COVID-19 patients [6, 9].

However, dp-ucMGP levels were significantly higher in ICU non-survivors than in survivors, after adjusting for age, gender, and APACHE II score. The average dp-ucMGP level over time was 167 pmol/L higher in ICU non-survivors (*p* < 0.05). Importantly, the results showed that the observed effect remained unchanged after adjustment for CRP, creatinine, history of coumarin use and also after adjustment for CAC. The latter seems to indicate that dp-ucMGP levels mark cardiovascular disease, which could play a role in ICU mortality in mechanically ventilated COVID-19 patients.

Interestingly, we observed that dp-ucMGP levels were higher at the moment of ETI and did not change over time in both survivors and non-survivors. When we consider only dp-ucMGP levels at ETI, higher levels were associated with higher OR for mortality, for both ICU and six month mortality in crude and adjusted modes, although not statistically significantly. Thus, the serial measurements showing a difference in dp-ucMGP on average over time between survivors and non-survivors, which is present at ETI, although higher dp-ucMGP levels at ETI itself appeared not statistically significantly associated to higher mortality and this is likely due to a type two error (lower power compared to serial dp-ucMGP analyses). These observations are consistent with previous reports of an association between increased inflammation and impaired extra-hepatic vitamin K levels [5, 7, 8]. The fact that dp-ucMGP levels remained lower over time in survivors compared to non-survivors suggests that extrahepatic vitamin K deficiency most likely marks disease severity, while a causal contribution to mortality itself is less likely.

Vitamin K is necessary for the activation of MGP, a protein that plays a key role in vascular calcification and arterial stiffness. Extra-hepatic vitamin K deficiency leads to the accumulation of dp-ucMGP, which has been linked to an increased risk of cardiovascular disease [2, 33–36]. Our study suggests that vitamin K deficiency, as indicated by elevated dp-ucMGP levels, may be particularly relevant in critically ill COVID-19 patients with pre-existing cardiovascular comorbidities.

Although our results suggest dp-ucMGP as a marker for disease severity previous studies alternatively have suggested that vitamin K itself plays a role in protecting against lung damage [37]. In COVID-19 in particular, some evidence suggests that vitamin K itself may also be relevant potentially acting in immune function and coagulopathy [6, 9]. The latter studies support the concept that vitamin K acts in severe cases of COVID-19 through immune dysregulation and coagulopathy which may lead to microvascular occlusion, multi-organ failure, and increased mortality rates.

The fact that dp-ucMGP levels remained stable over time in the current study does not necessarily contradict the assumption that vitamin K deficiency is associated with lung damage in COVID-19. It is important to note that we did not directly investigate the association between dp-ucMGP levels and COVID-19 or lung damage but rather assessed the levels of dp-ucMGP over time during ICU stay.

Our study had some limitations. First, because of a limited number of blood samples, we did not directly measure vitamin K levels; therefore, we cannot exclude the possibility that other factors may have contributed to the observed associations. Second, our study included only mechanically ventilated COVID-19 patients admitted to the ICU; therefore, our findings may not be generalizable to other patient populations. Finally, we did not investigate the effects of additional vitamin K supplementation on disease outcomes. The causal role vitamin K might play, could possibly be driven by nutrition and cannot be ruled out by our data. However, our manuscript provides more evidence that dp-ucMGP acts as a marker of cardiovascular disease instead of a causal factor. Additional adjustments for CAC did not change our results, however it is unclear if CAC acts as an adequate surrogate measure for cardio-vascular abnormalities in total.

## Conclusion

This study provides evidence that extrahepatic vitamin K deficiency, marked by high dp-ucMGP levels, occurs in COVID-19 ICU patients. ICU non-survivors have been shown to have higher dp-ucMGP levels over time, reflecting a more severe extrahepatic vitamin K deficiency which marks more cardiovascular disease. Our results, do neither support nor exclude the concept that vitamin K supplementation favours disease outcomes in COVID-19 patients.

## Data Availability

The datasets used and/or analysed during the current study are available from the corresponding author on reasonable request.

## Abbreviations

APACHE: Acute physiology and chronic health evaluation
aPTT: Activated partial thromboplastin time
BMI: Body mass index
CAC: Coronary artery calcification
COVID: Coronavirus disease
CRRT: Continuous renal replacement therapy
CTPA: Computed tomography pulmonary angiography
DOAC: Direct oral anticoagulants
dp-ucMGP: Dephosphorylated uncarboxylated matrix Gla protein
ECMO: Extracorporeal membrane oxygenation
ETI: Endotracheal intubation
HTR: Heparin therapeutic range
ICU: Intensive care unit
IQR: Interquartile range
LMWH: Low molecular weight heparin
MaastrICCht: Maastricht Intensive care covid
OR: Odds ratio
PE: Pulmonary embolism
PT: Prothombin time
RT-PCR: Real-time polymerase chain reaction
STROBE: Strengthening the reporting of observational studies in epidemiology
UFH: Unfractionated heparin
